# BERT-Based Neural Network for Inpatient Fall Detection From Electronic Medical Records: Retrospective Cohort Study

**DOI:** 10.2196/48995

**Published:** 2024-01-30

**Authors:** Cheligeer Cheligeer, Guosong Wu, Seungwon Lee, Jie Pan, Danielle A Southern, Elliot A Martin, Natalie Sapiro, Cathy A Eastwood, Hude Quan, Yuan Xu

**Affiliations:** 1 Centre for Health Informatics, Cumming School of Medicine University of Calgary Calgary, AB Canada; 2 Provincial Research Data Services Alberta Health Services Calgary, AB Canada; 3 Department of Community Health Sciences, Cumming School of Medicine University of Calgary Calgary, AB Canada; 4 Department of Oncology University of Calgary Calgary, AB Canada; 5 Department of Surgery University of Calgary Calgary, AB Canada

**Keywords:** accidental falls, electronic medical records, data mining, machine learning, patient safety, natural language processing, adverse event

## Abstract

**Background:**

Inpatient falls are a substantial concern for health care providers and are associated with negative outcomes for patients. Automated detection of falls using machine learning (ML) algorithms may aid in improving patient safety and reducing the occurrence of falls.

**Objective:**

This study aims to develop and evaluate an ML algorithm for inpatient fall detection using multidisciplinary progress record notes and a pretrained Bidirectional Encoder Representation from Transformers (BERT) language model.

**Methods:**

A cohort of 4323 adult patients admitted to 3 acute care hospitals in Calgary, Alberta, Canada from 2016 to 2021 were randomly sampled. Trained reviewers determined falls from patient charts, which were linked to electronic medical records and administrative data. The BERT-based language model was pretrained on clinical notes, and a fall detection algorithm was developed based on a neural network binary classification architecture.

**Results:**

To address various use scenarios, we developed 3 different Alberta hospital notes-specific BERT models: a high sensitivity model (sensitivity 97.7, IQR 87.7-99.9), a high positive predictive value model (positive predictive value 85.7, IQR 57.2-98.2), and the high *F*_1_-score model (*F*_1_=64.4). Our proposed method outperformed 3 classical ML algorithms and an International Classification of Diseases code–based algorithm for fall detection, showing its potential for improved performance in diverse clinical settings.

**Conclusions:**

The developed algorithm provides an automated and accurate method for inpatient fall detection using multidisciplinary progress record notes and a pretrained BERT language model. This method could be implemented in clinical practice to improve patient safety and reduce the occurrence of falls in hospitals.

## Introduction

### Background

Inpatient falls detrimentally impact patients, leading to extended hospital stays and distress among families and caregivers [1-5]. Studies reflect a varying incidence rate of such falls, with 250,000 annually in England and Wales alone [1], and evidence showing 7.5% of patients experience at least 1 fall during hospitalization [2]. Acute care hospitals also report a range of 1 to 9 falls per 1000 bed days, underscoring the pervasive nature of this problem [4]. Patients who fall may experience injuries that increase the risk of comorbidity or even disability [6,7]. They may also experience psychological effects such as anxiety, depression, or loss of confidence, which can affect their recovery and quality of life [8].

Manual chart review is regarded as one of the most common methods to identify inpatient falls [9]. This process involves the thorough examination of patient medical records to gather relevant information on the details of falls. Existing strategies include the Harvard Medical Practice Study [10] and the Global Trigger Tool [11]. Alternative methodologies, such as Patient Safety Indicators, based on International Classification of Diseases (ICD) codes, are used to identify adverse events (AEs), leveraging systematized health care data for detection [12-14]. However, these methodologies, while widely used, present challenges due to the time-consuming nature of ICD coding and manual chart reviews, potentially causing delays in recording and detecting AEs [15,16].

Free text data in electronic medical records (EMRs) offer rich, up-to-date insights into patients’ health status, medications, and various narrative content. Despite its wealth of information, the unstructured nature of this data necessitates chart reviews, a labor-intensive process, to identify inpatient falls [17]. There has been an increasing interest in recent years in applying natural language processing (NLP) techniques to electronic clinical notes to automate disease identification and create clinical support decision systems [18-24].

Previous NLP studies in the detection of patient fall including rule-based algorithms [25,26] and machine learning (ML) methods [27-30] have been explored, but they often struggle with the variety and complexity of clinical language.

The deep learning model Bidirectional Encoder Representation from Transformers (BERT) [31] can effectively address these challenges. It uses transformer architecture to understand text contextually, handling linguistic complexity, abbreviations, and data gaps, thereby augmenting text understanding from EMR [20]. The use of transformer-based methods to understand EMR text data has emerged as a promising new trend in automatic clinical text analysis [32].

### Objectives

In this study, we intend to pretrain an existing model, BioClinical BERT [33], with free text data from Alberta hospital EMRs to develop an Alberta hospital notes-specific BERT model (AHN-BERT). The pretrained language model would serve as a feature extraction layer in a neural network to identify inpatient falls. We hypothesize that fine-tuning BERT on local hospital data will enable more accurate fall detection compared with generic models. Additionally, we expect AHN-BERT will outperform conventional rule-based and ML approaches, as well as ICD code methods, in detecting falls from unstructured EMR notes in near real time. By evaluating AHN-BERT against current techniques, we hope to demonstrate the value of transfer learning with BERT for improved efficiency and generalizability in surfacing patient safety events from clinical text. Ultimately, our goal is to advance the detection of inpatient falls, allowing for more detailed and accurate patient safety interventions. An improved fall detection system could potentially enable health care providers to swiftly implement preventive measures, reducing the incidence and severity of falls. Additionally, through the facilitation of access and analysis of fall-related data, our system could become an invaluable resource for researchers investigating fall prevention and associated subjects.

## Methods

### Overview

In our methodology, we emphasized a detailed and transparent approach, covering all aspects from data collection to model validation. This comprehensive process, reflecting best practices in research reporting [34], ensures clarity and precision in our multivariable prediction model, providing an in-depth understanding of its performance and applicability.

### Source of Data

Our study is a retrospective analysis. We used a stratified random sample of adult patients admitted to acute care hospitals in Calgary, Alberta. We linked the extracted EMR data to Sunrise Clinical Manager (SCM) records and ICD-coded discharge abstract database (DAD) using an established mechanism [35]. Both tables are stored and managed by the Oracle database.

The chart reviewer team consists of 6 registered nurses with 1 to 10 years of experience using SCM for clinical care. The nurses followed a training procedure, and 1 trained nurse became the project lead for quality assurance. The training involved learning the condition definitions and practicing reviewing each chart systematically. Reviewers examined the entire record for specified conditions and consulted each other with questions. In the process of training and quality assurance, we tested interrater reliability using Conger, Fleiss, and Light κ methods, with 2 nurses reviewing the same set of 10 charts for consensus on AEs. Where agreement was poor (κ<0.60), retraining occurred until high agreement (κ>0.80) was achieved [36]. Reviewers then proceeded independently with REDCap tool (Vanderbilt University).

The chart review data served as the reference standard to develop and evaluate our fall detection model. We focused on multidisciplinary progress records (MPRs) for fall detection, as chart review data showed most falls (115/155, 73.7%) were documented in MPRs by nursing staff. We created supervised data sets for the classification task to identify optimal fall detection timing, including 1-day (fall day MRPs notes), 2-day (fall day + day after), 3-day (fall day + 2 days after), and full hospitalization MRPs. All supervised data sets were labeled to indicate whether notes were associated with inpatient falls. For the training of our model, we used both cases (falls) and controls (nonfalls) at a ratio of 1:29. This was done to ensure the model was exposed to a balanced representation of both scenarios. Our test set mirrored the real-world data distribution to enable an accurate evaluation of model performance. In addition, we constructed an unsupervised corpus specifically for language model pretraining. This corpus comprises free-text note data and does not rely on any predetermined labels or annotations.

### Participants

At the time of the study, a total of 4393 charts were reviewed, among which we identified a total of 155 records as falls and 4238 records as no falls. The study included only the first admission of each patient, even if they had multiple hospitalizations within the study period. We exclusively focused on adults 18 to 100 years of age, thereby excluding minors and centenarians. Furthermore, if a patient had multiple fall incidents, only the most recent record was considered, although no such cases were identified during the study. The temporal framework for the study encompassed a decade, from 2010 to 2020. Exclusion criteria were also clearly defined: patients without unstructured note data or those who could not be linked using our established data linkage mechanism were omitted from the study.

### Missing Data and Data Cleaning

Our study implemented rigorous data cleaning to ensure data integrity. After conducting a conflict review and excluding records with inconsistencies in fall status documentation (17 records checked for both falls and no falls), failed data linkage (1 record), temporal conflicts between fall and admission dates (5 records), and missing MPR documentations (47 records), the final clean data set totaled 4323 records (142 falls and 4181 no falls).

### Outcome and Variables

The desired outcome of our proposed framework is to predict whether a patient’s daily progress note contains hints about inpatient falls. The input to our model is each patient’s n-day note. We use a BERT model to represent the textual data in numerical format, also known as contextualized word embeddings.

The input text is represented by 768-dimensional feature vectors, which can be considered as 768 variables. However, due to the distributed representation of neural language models, each variable does not represent a single word. Instead, individual variables preserve contextual information segments for each word, constituting meaningful vector representations of the entire input text.

On a related note, we have also collected and analyzed several demographic and clinical variables for our patient cohort from DAD database. Although not directly used in our predictive modeling, these variables furnish invaluable insights into the characteristics of our study population and contribute to the overall richness of our research data. These include age at the time of admission, sex, the incidence of intensive care unit visits during the hospital stays, the length of the hospital stays, and the hospital’s geographical location. The latter was particularly focused on 3 acute care hospitals based in Calgary, Alberta: hospitals “A,” “B,” and “C.” These variables help us understand the context in which the patient notes were written and may influence the interpretation of the model’s results.

### Sample Size

We included all patient data that has been reviewed by the reviewer team and filtered out from the inclusion-exclusion criteria.

### Model Development

To conform to the BERT input length limit, MPR notes exceeding 400 tokens were programmatically split into segments under 400 words, preserving contextual information. All notes underwent preprocessing including removal of extraneous headers, signatures, whitespaces, and escape characters using regular expressions, and duplicate sentences were eliminated.

**Figure 1:**
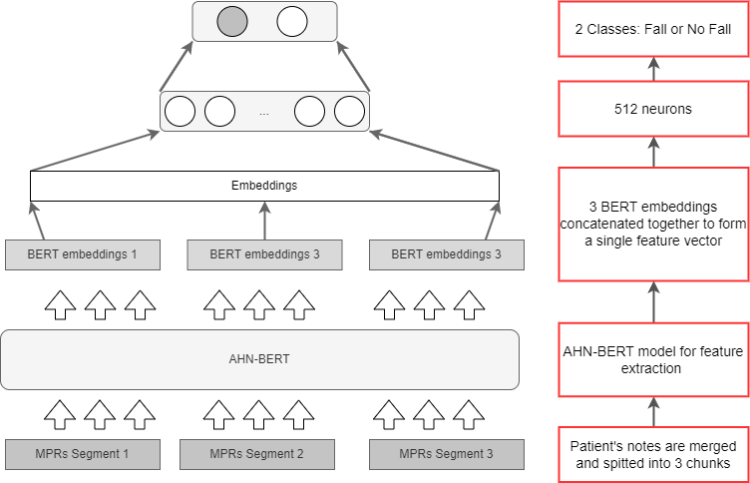
Proposed model architecture. AHN-BERT: Alberta hospital notes-specific BERT; BERT: Bidirectional Encoder Representation from Transformers; MPR: multidisciplinary progress record.

Our model architecture comprises 2 key components, an Alberta hospital note-specific BERT model for contextual feature extraction from clinical text, which we term AHN-BERT, and a feedforward neural network classifier to detect falls from the extracted features (as Figure 1).

AHN-BERT was initialized with weights from BioClinical BERT and further pretrained on our corpus of unsupervised hospital notes to adapt to local clinical terminology and language patterns. To prevent bias from overly lengthy documents, notes were filtered to be between 50 and 5000 tokens prior to pretraining. AHN-BERT was pretrained using a masked language modeling technique on 15% of randomly selected input tokens, enabling learning of contextual representations of clinical text without explicit labels. For feature extraction, AHN-BERT processes up to 3 concatenated note segments under 400 tokens. The resulting “[CLS]” vectors summarizing the semantic content of each segment are aggregated via concatenation to represent the full note’s contextual information [37].

A feedforward neural network is used as the classifier, taking the concatenated features as input. The network comprises fully connected layers to map the features into class probabilities for fall detection. Dropout regularization is implemented in the classifier to prevent overfitting to the training data. Sigmoid activation in the output layer provides posterior probabilities for the binary fall classification task.

### Model Assessment

First, to determine the optimal timeframe for note selection that best represents inpatient falls, we compared model performance using 1-day, 2-day, 3-day, and complete patient note data sets. Since the exact time lapse between an inpatient fall and corresponding documentation is variable, we evaluated these distinct time intervals in a data-driven approach to identify the optimal period for note selection. We used the same model architecture and pretrained AHN-BERT for all data sets, comparing training and validation loss convergence and evaluation metrics to assess performance.

Second, we tuned the classification probability threshold to balance model sensitivity and precision. The threshold denotes the cutoff for determining class membership based on predicted probabilities. By optimizing the threshold, we controlled the tradeoff between correctly identifying true positives and avoiding false positives. We developed three distinct models by threshold tuning for different purposes: (1) a high-sensitivity model that maximizes sensitivity by optimizing the threshold, (2) a high positive predictive value (PPV) model that maximizes PPV through threshold optimization, and (3) a high *F*_1_-score model that balances sensitivity and PPV by optimizing the threshold, serving as a general-purpose model.

Third, we conducted a comparative evaluation between our top-performing neural network model and several other approaches, including 2 alternative BERT-based models, 3 conventional ML models, and an ICD-code–based algorithm. The 2 additional BERT-based models used original pretrained BERT and BioClinical BERT as feature extractors. For the 3 conventional ML models (support vector machine, logistic regression, and decision tree classifiers), we used bag-of-words features and term frequency-inverse document frequency weighting. These models were trained and compared on the 1-day MPRs data set. The ICD-code algorithm was applied to the same patient cohort but relied on administrative diagnosis codes rather than clinical notes. It aimed to demonstrate the efficacy of standard diagnostic codes for identifying falls compared with our neural network model. Falls were identified by the presence of ICD-10 codes W00-W20 when not listed as the primary diagnosis.

### Statistical Analysis

The characteristics of the patients included in the study were thoroughly evaluated. These characteristics encompassed age, sex, the incidence of intensive care unit visits, the length of their hospital stay, and their originating hospitals. We summarized categorical variables as frequencies and percentages, while continuous variables were expressed as medians and IQRs. The *χ*^2^ test was used for categorical variables to determine statistical differences, while the Wilcoxon rank-sum test was used for continuous variables. A *P* value threshold of 5% or lower was set to denote statistical significance.

To evaluate our ML model, we calculated several statistical metrics such as sensitivity, specificity, PPV, negative predictive value, accuracy, and *F*_1_-score.

### Computational Environment

Our study harnessed a high-performance computing environment, primarily driven by an NVIDIA GeForce RTX 3080 GPU with 16GB of memory, vital for pretraining and fine-tuning our language model. The statistical analysis and experiment leveraged Python 3.8, and libraries such as NumPy [38], Scikit-learn [39], Pandas [40], and PyTorch [41] for tasks like data processing and modeling.

### Ethics Approval

This study was approved by the Conjoint Health Research Ethics Board at the University of Calgary (REB21-0416). Patient consent was waived as part of the ethics board review process.

## Results

### Participants

Our final study cohort contains 4323 individuals, with 142 (3.28%) patients identified by chart reviewers as having falls recorded in their medical charts during their hospital stay. The remaining 4181 (96.7%) did not fall. All patients were successfully linked to the SCM and DAD by unique identification number and admission date. Table 1 presents the descriptive statistics in general. Multimedia Appendix 1 further stratifies Table 1 into respective hospitals (Multimedia Appendix 1).

**Table 1:** Descriptive statistics of the study cohort.

		Total (n=4323)	Confirmed fall (n=142)^a^	No fall (n=4181)^b^	*P* value^c^	
Age, median (IQRs^d^)		62.0 (48.5-75.5)	71.0 (59.6-82.4)	61.0 (47.5-74.5)	<001	
Sex (male), n (%)		2169 (50.2)	73 (51.4)	2096 (50.1)	.77	
ICU^e^ visit, n (%)		163 (3.8)	19 (13.4)	144 (3.4)	<.001	
Length of hospital stay (days), median (IQRs)		3.0 (0.5-5.5)	12.0 (1.5-22.5)	3.0 (0.5-5.5)	<.001	
**Hospitals, n (%)^f^**						.04
	Hospital “A”	3548 (82.1)	104 (73.2)	3444 (82.4)		
	Hospital “B”	651 (15.1)	34 (23.9)	617 (14.8)		
	Hospital “C”	124 (2.8)	4 (2.9)	120 (2.8)		

^a^A term used in the study to refer to patients who fell during their hospital stay and were confirmed to have fallen through medical records or other documentation.

^b^A term used in the study to refer to patients who did not fall during their hospital stay.

^c^A measure indicating the statistical significance (*P*<.05) of the observed difference between groups.

^d^A measure of statistical dispersion representing the difference between the 75th and 25th percentiles of a data set.

^e^ICU: intensive care unit.

^f^Three different hospitals were included in the study: hospitals A, B, and C.

### Model and Framework Assessment

First, to determine the optimal timeframe, we compared 1-day, 2-day, 3-day, and complete note data sets using the same model architecture and AHN-BERT pretrained embeddings. We trained each model for 200 epochs, with the primary goal of comparing their overall performance on the test sets. Evaluating performance metrics and training convergence, the 1-day data set was most effective and efficient, achieving 93.0% sensitivity and 83.0% specificity.

Second, we optimized the classification threshold to balance sensitivity and precision. These models maximize sensitivity, PPV, and *F*_1_-score respectively. As results are shown in Table 2, our proposed architecture with AHN-BERT achieved overall the highest metrics among the comparison.

Third, the comparative assessment showed our approach outperformed 2 alternative BERT models, 3 classical ML models (support vector machine, logistic regression, and decision tree), and an ICD-code algorithm. The BERT models used original BERT and BioClinical BERT embeddings, while the ML models used bag-of-words and term frequency-inverse document frequency on the 1-day data set. The ICD method relied on administrative codes rather than text. Our neural network model demonstrated superior inpatient fall detection across different methods and data sources.

Our high sensitivity model exhibited 97.7% sensitivity, enabling near-perfect capture of relevant notes, along with 82.3% accuracy, but a low 26.8% *F*_1_-score. The high PPV model achieved 97.5% accuracy, 85.7 % PPV, and 27.9 % sensitivity. The high *F*_1_-model balanced 66.7% sensitivity and 60.5% PPV to optimize 64.4% *F*_1_-score and 97.7% accuracy. In comparison, the ICD-based method had 27.9% sensitivity, while traditional classifiers achieved 51.2%-76.7% sensitivities and 8.3-15.8 PPVs.

**Figure 2:**
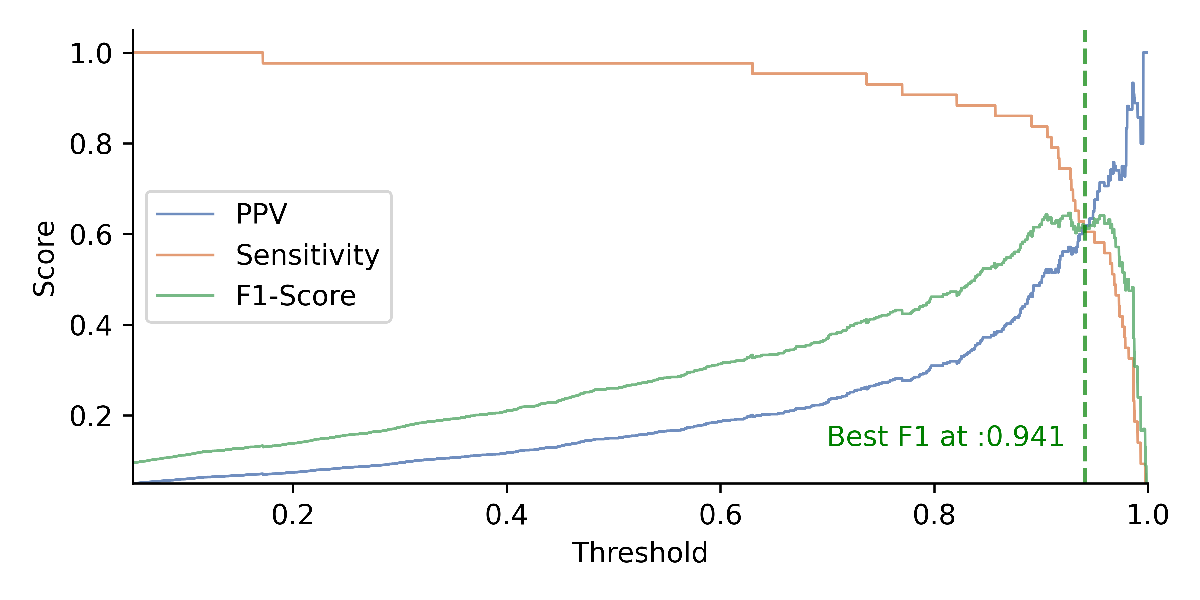
Performance metrics at varying thresholds: PPV, sensitivity, and F1-score. PPV: positive predictive value.

**Figure 3:**
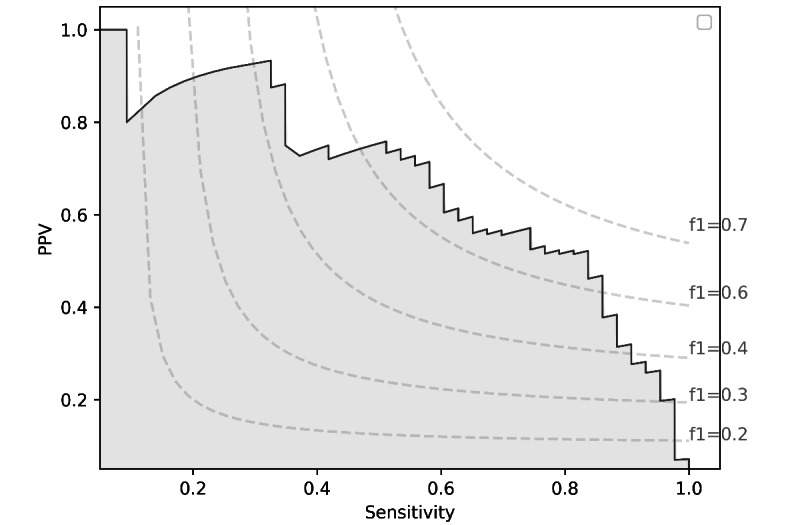
Percision-recall curve. PPV: positive predictive value.

The result of the probability-based threshold adjustment in accordance with PPV, sensitivity, and *F*_1_-score is shown in Figure 2. By adjusting the classification threshold, we can control the trade-off between sensitivity and precision (as Figure 3).

**Table 2:** Performance of proposed deep learning models, classical machine learning methods, and International Classification of Diseases–based algorithms on fall identification with 1-day data set.

Category and model name		Sensitivity (%), (95% CI)	Specificity (%), (95% CI)	PPV^a^ (%), (95% CI)	NPV^b^ (%), (95% CI)	Accuracy (%), (95% CI)	*F*_1_-score^c^ (%)
**BERT^d^-based models**							
	AHN-BERT^e^ (high sensitivity)	97.7 (87.7-99.9)	81.8 (79.6-83.9)	15.6 (14.0-17.3)	99.9 (99.3-100.0)	82.3 (80.1-84.4)	26.8
	AHN-BERT (high PPV)	27.9 (15.3-43.7)	99.8 (99.4-100.0)	85.7 (57.2-98.2)	97.6 (96.6-98.4)	97.5(96.5-98.2)	42.1
	AHN-BERT (high *F*_1_)	66.7 (49.8-80.9)	99.0 (98.2-99.5)	60.5 (44.4-75.0)	98.7 (97.8-99.2)	97.7 (96.7-98.4)	63.4
	BERT-uncased	79.1 (64.0-9 0.0)	61.4 (58.6-64.1)	6.6 (5.6-7.7)	98.8 (97.9-99.4)	62.0 (59.3-64.6)	12.1
	BioClinical BERT	74.4 (58.8-86.5)	69.8 (67.2-72.4)	7.8 (6.5-9.3)	98.8 (97.9-99.3)	70.0 (67.4-72.5)	14.1
**Classical machine learning classifier**							
	Support vector machine	76.7 (61.4-88.2)	85.0 (82.9-86.9)	14.9 (12.5-17.8)	99.1 (98.4-99.5)	84.7 (82.6-86.6)	25.0
	Logistic regression	74.4 (58.8-86.5)	86.4 (84.3-88.2)	15.8 (13.0-19.0)	99.0 (98.3-99.4)	86.0 (83.9-87.8)	26.0
	Decision tree	51.2 (35.5-66.7)	93.9 (92.4-95.1)	22.2 (14.5-31.7)	98.3 (97.3-98.9)	92.4 (90.9-93.8)	31.0
**Rule-based classifier**							
	ICD 10^f^	27.9 (15.3-43.7)	92.3 (90.7-93.8)	11.1 (6.9-17.3)	97.4 (96.9-97.8)	90.2 (88.5-91.8)	15.9

^a^PPV: positive predictive value. The proportion of true positive results among all positive results.

^b^NPV: negative predictive value. The proportion of true negative results among all negative results.

^c^*F*_1_-score: a measure of a model’s accuracy that considers both sensitivity and PPV.

^d^BERT: Bidirectional Encoder Representations from Transformers.

^e^AHN-BERT: Alberta hospital notes-specific BERT.

^f^ICD 10: International Classification of Diseases, 10th Revision.

## Discussion

### Principal Results

In our study, we illustrate how a BERT-based model substantially outperforms the ICD-based algorithm in fall detection within the hospital setting. This superiority stems from the model’s ability to process EMR text data in real time, enabling rapid identification of falls. In contrast, ICD codes are assigned retrospectively, leading to delays in fall detection and intervention. Using BERT’s advanced NLP facilitates accurate, efficient, and generalizable analysis of clinical notes for surveillance applications.

Specifically, our proposed AHN-BERT model surpasses generic BERT, conventional ML, and ICD-codes. Fine-tuning BERT on local hospital notes better captures local domain-specific language and context, boosting performance. Additionally, combining high-sensitivity and high-PPV models enables optimized 2-stage fall detection by adjusting decision thresholds to balance false positives and negatives. This provides flexibility for different use cases and challenging tasks.

Furthermore, our study provides valuable insights into the optimal time frame for defining falls empirically. This comparison sheds light on the potential benefits of using a finer-grained time interval, which could improve the generalizability and applicability of the model across different populations and settings. Understanding the optimal period for detecting fall incidents can guide the development and implementation of targeted public health interventions. Health surveillance data can be used to evaluate the effectiveness of these interventions and inform future strategies for fall prevention and management. By determining the most suitable time interval for defining fall incidents, health surveillance systems can better allocate resources to areas with a higher risk of falls. This may result in more efficient and effective public health efforts, improving health outcomes for at-risk populations.

### Applications

Our models leverage unstructured EMR data to accurately detect inpatient falls, enabling health care systems to enact tailored prevention measures and reduce fall-associated injuries. The automation of extensive clinical documentation review accelerates health care surveillance and quality improvement processes.

Regarding research applications, our algorithms can extract comprehensive fall data from EMR text to support developing evidence-based interventions.

The proposed framework has broad applicability beyond fall detection for tasks like diagnosis prediction, medication adherence monitoring, and adverse drug event identification. This adaptability improves health care outcomes, patient safety, and quality of care.

### Strength and Limitations

In our research, the AHN-BERT model has shown remarkable superiority over traditional ICD-based algorithms in fall detection within hospital environments. This enhanced performance is primarily attributed to the model’s proficiency in processing and understanding the nuances of EMRs text data. Unlike ICD codes, which can sometimes result in undercoding or loss of information, the nursing notes processed by our model are more closely aligned with the actual circumstances of inpatient falls. The ability of AHN-BERT to immediately and accurately process this data is a substantial advancement, ensuring that fall detection is not only more precise but also more reflective of the true clinical scenario. Additionally, the combination of high-sensitivity and high-PPV models in our 2-stage fall detection system allows for adjustable decision thresholds, thus balancing false positives and negatives and providing flexibility across different scenarios.

However, the model faces challenges in balancing high sensitivity with a high PPV due to the imbalanced nature of clinical data. The rarity of AEs like falls leads to a higher rate of false positives, as seen in our data set with a significant imbalance ratio. Our test data set, characterized by a significant 29:1 imbalance, aligns more closely with real-world clinical scenarios than balanced data sets used in some prior studies [27], which, while yielding promising results, may not fully represent practical conditions. This intentional choice ensures that our model’s performance is tested under conditions typical of rare events like falls, thereby enhancing its relevance and utility in actual clinical settings.

Second, the effectiveness of our models depends on the quality and comprehensiveness of documentation. If fall events or associated risk factors are not well documented, our model, like any data-driven model, may have difficulty detecting them. This underscores the importance of careful, detailed clinical documentation to enhance the effectiveness of monitoring applications. In addition, our study also assumes a certain level of linguistic and terminological consistency within the EMR data. Variations in documentation styles across different health care providers could potentially impact the model’s performance, suggesting that future models should incorporate strategies, for example, pretraining the ML, to mitigate such discrepancies. Last, the differentiation between a history of falls and inpatient falls presents a challenge, as it could potentially lead to false positive predictions if falls that occurred prior to hospitalization are documented in the notes. Although the BERT model’s contextual understanding can partially alleviate this issue, we acknowledge that more improvements are needed. As part of our future work, we aim to further refine our model to better handle such complexities.

### Conclusions

This study developed and evaluated BERT-based NLP models for the automated detection of falls from electronic clinical notes. The developed models provided a more accurate and timely way to detect falls than traditional ML and ICD-codes–based methods. Moreover, we provided a masked language model technique to pretrain a pre-existing BERT model using clinical text data gathered from various health care facilities in Calgary, Alberta, creating a more local institution-specific and effective AHN-BERT model. By using self-supervised language modeling strategies, we can bypass steps that were regarded as vital in standard ML methods, such as the necessity for thorough text preprocessing, complex feature engineering, and a considerable amount of labeled data. In addition, by exploring the optimal period for fall incident detection and selecting 1-day notes for our final architecture, our model contributes to enhanced patient safety and care with less noise.

## Appendix

Multimedia Appendix 1 Descriptive statistics for the study cohort from each hospital.

## Abbreviations

AE: adverse event
AHN-BERT: Alberta hospital notes-specific BERT
BERT: Bidirectional Encoder Representation from Transformers
DAD: discharge abstract database
EMR: electronic medical record
ICD: International Classification of Diseases
ML: machine learning
MPR: multidisciplinary progress record
NLP: natural language processing
PPV: positive predictive value
SCM: Sunrise Clinical Manager
