# Supplementary material for: BERT-Based Neural Network for Inpatient Fall Detection From Electronic Medical Records: Retrospective Cohort Study
Source: JMIR Med Inform. 2024 Jan 30;12:e48995. doi: 10.2196/48995 (PMC10865188; doi:10.2196/48995)
Supplement: Multimedia Appendix 1 [file medinform_v12i1e48995_app1.docx]

Multimedia appendix 1. Descriptive statistics for the study cohort from each hospital.

| **Hospital A** | | Total (n=3548) | Confirmed Fall (n=104) | | No Fall (n=3444) | | *P*-value |
| --- | --- | --- | --- | --- | --- | --- | --- |
| **Age, Median (IQRs)** | | 61.0 (48.0-74.0) | 68.0 (57.0-79.0) | | 61.0 (47.88-74.12) | | <.001 |
| **Sex, Male** | | 1829 (51.6%) | 54 (51.9%) | | 1775 (51.5%) | | =0.94 |
| **ICU Visit** | | 138 (3.9%) | 18 (17.3%) | | 120 (3.5%) | | <.001 |
| **Length of hospital stay (days), Median (IQRs)** | | 3.0 (0.5-5.5) | 12.5 (1.88-23.12) | | 3.0 (0.5-5.5) | | <.001 |
|  | | | | | | | |
| **Hospital B** | | Total (n=651) | Confirmed Fall (n=34) | | No Fall (n=617) | | *P*-value |
| **Age, Median (IQRs)** | | 67.0 (49.5-84.5) | 81.5 (72.25-90.75) | | 66.0 (48.5-83.5) | | <.001 |
| **Sex, Male** | | 288 (44.2%) | 17 (50.0%) | | 271 (43.9%) | | =0.52 |
| **ICU Visit** | | 17 (2.6%) | 1 (2.9%) | | 16 (2.6%) | | =0.91 |
| **Length of hospital stay (days), Median (IQRs)** | | 4.0 (1.0-7.0) | 12.5 (2.75-22.25) | | 4.0 (1.0-7.0) | | =0.002 |
|  | | | | | | | |
| Hospital C | Total (n=124) | | | Confirmed Fall (n=4) | | No Fall (n=120) | *P*-value |
| **Age, Median (IQRs)** | 57.0 (40.38-73.62) | | | 89.5 (84.12-94.88) | | 56.0 (40.5-71.5) | =.0012 |
| **Sex, Male** | 52 (41.9%) | | | 2 (50.0%) | | 50 (41.7%) | =0.79 |
| **ICU Visit** | 8 (6.5%) | | | 0 (0.0%) | | 8 (6.7%) | =0.004 |
| **Length of hospital stay (days), Median (IQRs)** | 3.0 (0.5-5.5) | | | 8.5 (2.0-15.0) | | 3.0 (0.5-5.5) | =0.43 |

**^a^IQR:** Interquartile range, a measure of statistical dispersion representing the difference between the 75th and 25th percentiles of a data set.

**^b^ICU:** Intensive Care Unit.

**^c^P-value:** A measure indicating the statistical significance (P<0.05) of the observed difference between groups.

**^d^Hospital A, B, C**: Three different hospitals were included in the study.

**^e^Fall:** A term used in the study to refer to patients who fell during their hospital stay and were confirmed to have fallen through medical records or other documentation.

**^f^No Fall:** A term used in the study to refer to patients who did not fall during their hospital stay.

**^g^LOS**: Length of stay, measured in days, a metric measuring the number of days spent in the hospital.
